# Supplementary material for: Perianal Structures in Non-Myrmecophilous Aphids (Hemiptera, Aphididae)
Source: Insects. 2023 May 16;14(5):471. doi: 10.3390/insects14050471 (PMC10231127; doi:10.3390/insects14050471)
Supplement: Supplementary file 1 [file insects-14-00471-s001.zip › insects-2365261-supplementary.pdf]

Supplementary material Table S1. Measurements of perianal structures.

Species	Anal plate length (mm)	Anal plate width in the widest place	Cauda length	Width cauda at the base	Half width cauda
Drepanosiphum platanoidis	0.109	0.176	0.170	0.132	0.039
	0.125	0.239	0.158	0.182	0.079
	0.085	0.200	0.199	0.229	0.068
	0.188	0.187	0.189	0.248	0.061
	0.112	0.240	0.208	0.192	0.090
Clethrobius comes	0.109	0.319	0.161	0.241	0.99
	0.111	0.361	0.233	0.236	0.108
	0.113	0.348	0.194	0.242	0.84
	0.092	0.333	0.206	0.215	0.135
	0.137	0.156	0.139	0.210	0.100
Myzocallis coryli	0.112	0.160	0.132	0.115	0.089
	0.083	0.157	0.089	-	0.063
	0.083	0.152	0.087	0.128	0.47
	0.082	0.152	0.125	0.130	0.064
	0.086	0.163	0.90	0.135	0.065
Myzocallis carpini	0.074	0.141	0.114	0.113	0.038
	0.089	0.118	0.107	0.102	0.045
	0.092	0.155	0.106	0.120	0.075
	0.102	0.164	0.111	0.132	0.089
	-	-	-	-	-
Tinocallis takachihoensis	0.063	0.165	0.063	0.790	0.043
	0.071	0.196	0.094	0.090	0.055
	0.078	0.188	0.102	0.118	0.55
	0.071	0.157	0.086	0.087	0.063
	0.094	0.196	0.11	0.141	0.071
Euceraphis betulae	0.147	0.253	0.253	0.177	0.067
	-	0.264	0.246	0.211	0.085
	0.118	0.245	0.252	0.219	0.076
	0.097	0.185	0.246	0.198	0.087
	0.224	0.337	0.235	0.202	0.094
Mimeuria ulmiphila	0.079	0.220	0.039	0.181	0.118
	0.102	0.181	0.039	0.141	0.102
	-	-	-	-	-
	-	-	-	-	-
	-	-	-	-	-
Tetraneura ulmi	0.089	0.302	0.095	0.153	0.072
	-	0.200	0.112	0.174	0.111
	0.09	0.175	0.049	0.107	0.065
	0.139	0.182	0.049	0.168	0.075

	0.131	0.162	0.041	0.103	0.061
Eriosoma ulmi	0.245	0.245	0.076	0.233	0.129
	0.111	0.183	0.043	0.131	0.095
	-	-	-	-	-
	-	-	-	-	-
	-	-	-	-	-
Baizongia pistaciae	0.098	0.196	0.078	0.194	0.140
	0.106	0.211	0.076	0.221	0.153
	0.116	0.198	0.138	0.316	0.206
	-	-	-	-	-
	-	-	-	-	-
Cinara (Schizolachnus) pineti	0.072	0.192	0.083	0.119	0.078
	0.072	0.184	0.082	0.185	0.106
	0.118	0.272	0.100	0.223	0.142
	0.104	0.329	0.130	0.338	0.154
	0.109	0.250	0.118	0.0333	0.132
Tuberolachnus salignus	0.246	0.554	0.138	0.369	0.261
	0.215	0.569	0.146	0.385	0.292
	0.215	0.615	0.108	0.384	0.308
	0.184	0.569	0.153	0.369	0.292
	-	-	-	-	-
Brevicoryne brassicae	0.086	0.204	0.126	0.145	0.078
	0.078	0.236	0.157	0.173	0.102
	0.102	0.181	0.149	0.134	0.077
	-	-	-	-	-
	-	-	-	-	-
Cavariella theobaldi	0.071	0.196	0.165	0.117	0.086
	0.078	0.204	0.165	0.102	0.094
	0.056	0.189	0.118	0.094	0.086
	0.063	0.204	0.157	0.102	0.094
	-	-	-	-	-
Cavariella pastinacae	0.055	0.173	0.149	0.086	0.078
	0.059	0.165	0.118	0.079	0.071
	0.063	0.157	0.149	0.071	0.063
	-	-	-	-	-
	-	-	-	-	-
Corylobium avellanae	0.205	0.268	0.228	0.184	0.87
	0.189	0.237	0.226	0.173	0.087
	-	-	0.203	0.286	0.108
	0.178	0.261	0.229	0.207	0.083
	-	-	-	-	-
Liosomaphis berberidis	0.145	0.266	0.178	0.157	0.124
	0.102	0.289	0.207	0.143	0.116
	-	0.275	0.196	0.162	0.120
	0.123	0.277	0.201	0.167	0.115
	0.111	0.283	0.187	0.126	0.128
Uroleucon cichorii	0.187	0.389	0.785	0.302	0.168

	0.153	0.391	0.770	0.265	0.189
	0.153	0.391	0.608	0.245	0.155
	0.170	0.340	0.646	0.258	0.149
	0.161	0.356	0.604	0.230	0.146
Uroleucon aeneum	0.185	0.348	0.534	0.220	0.119
	0.238	0.368	0.563	0.213	0.144
	0.120	0.312	0.312	0.200	0.085
	0.161	0.285	0.574	0.193	0.124
	0.184	0.384	0.728	0.244	0.156
Uroleucon jaceae	0.212	0.432	0.786	0.212	0.196
	0.227	0.353	0.723	0.251	0.188
	0.173	0.299	0.707	0.188	0.196
	0.149	0.306	0.534	0.181	0.134
	0.157	0.338	0.613	0.196	0.141
Eulachnus nigricola	0.039	0.196	0.047	0.173	0.118
	0.024	0.149	0.039	0.141	0.086
	0.055	0.188	0.047	0.173	0.118
	0.031	0.165	0.055	0.149	0.094
	-	-	-	-	-
Macrosiphoniella artemisiae	0.145	0.364	0.540	0.206	0.149
	0.155	0.283	0.528	0.193	0.153
	0.179	0.389	0.515	0.213	0.180
	0.172	0.380	0.517	0.216	0.195
	0.145	0.271	0.493	0.161	0.145
Macrosiphoniella pulvera	0.150	0.244	0.346	0.141	0.134
	0.102	0.244	0.361	0.126	0.094
	0.157	0.275	0.353	0.110	0.118
	0.118	0.251	0.416	0.126	0.118
	-	-	-	-	-
Macrosiphoniella tanacetaria	0.169	0.234	0.521	0.187	0.135
	0.147	0.284	0.530	0.185	0.128
	0.161	0.345	0.496	0.203	0.141
	0.167	0.324	0.503	0.214	0.157
	0.175	0.304	0.567	0.210	0.193
Prociphilus fraxinifolii	0.108	0.250	0.125	0.297	0.2
	0.265	0.263	0.084	0.137	0.099
	0.334	0.420	0.147	0.321	0.189
	0.166	0.247	0.078	0.250	0.116
	-	-	-	-	-
	-	-	-	-	-
